# The Proposed Future Infrastructure Model for Basic Occupational Health Services in Malaysia

**DOI:** 10.21315/mjms2019.26.2.14

**Published:** 2019-04-30

**Authors:** Niza Samsuddin, Ailin Razali, Nor Azlina Ab Rahman, Muhammad Zubir Yusof, Nik Ahmad Kamal Nik Mahmood, Ahmad Fitri Abdullah Hair

**Affiliations:** 1 Department of Biomedical Science, Kulliyyah of Allied Health Sciences, International Islamic University Malaysia, Bandar Indera Mahkota, Kuantan, Pahang, Malaysia; 2 Department of Occupational Safety, Health and Built Environment, Mahallah Maimunah Office, International Islamic University Malaysia, Bandar Indera Mahkota, Kuantan, Pahang, Malaysia; 3 Department of Otolaryngology - Head & Neck Surgery, Kulliyyah of Medicine, International Islamic University Malaysia, Bandar Indera Mahkota, Kuantan, Pahang, Malaysia; 4 Department of Community Medicine, Kulliyyah of Medicine, International Islamic University Malaysia, Bandar Indera Mahkota, Kuantan, Pahang, Malaysia; 5 Department of Civil Law, Ahmad Ibrahim Kulliyyah of Laws, International Islamic University Malaysia, Gombak, Selangor, Malaysia; 6 Occupational Health Division, Department of Occupational Safety & Health, Level 4, Block D4, Complex D, Federal Government Administrative Centre, W. P. Putrajaya, Malaysia

**Keywords:** basic occupational health services, occupational health services, small and medium enterprises, infrastructure model

## Abstract

The objectives of occupational health services (OHS) are to create a healthy and safe working environment, prevent work-related diseases, optimise employees’ functional capacity and promote health. According to the literature, global accessibility to OHS has not shown much improvement and even worsened in certain countries. The main challenges come from the small and medium enterprises (SMEs). To respond to these global challenges, the basic occupational health services (BOHS) guideline was published under the purview of the World Health Organization and the International Labour Organization. The guideline describes BOHS as part of the infrastructure called the occupational safety and health system, an essential element that ensures the high service coverage and sustainability of the programme. The BOHS guideline was introduced in Malaysia by the Department of Occupational Safety and Health with a focus on SMEs, but its accessibility is low. A gap analysis was conducted between the current BOHS in Malaysia and the published international guideline. The important challenges identified that contributes to the low BOHS accessibility in Malaysia is the weakness in the BOHS infrastructure and OHS system provision. The proposed BOHS infrastructure model is meant to increase accessibility and to provide fair and equitable health services for Malaysians.

## Introduction

The International Labour Organization/World Health Organization (ILO/WHO) joint committee was formed to deliberate on occupational health services (OHS). The committee found that the achievements of OHS in many countries were inadequate, although efforts have been made globally and in developed countries, especially among workers in small and medium enterprises (SMEs). To meet the global needs, a joint committee of the ILO/WHO developed the concept of basic occupational health services (BOHS) in 2003. In response to the new BOHS concept, the WHO, ILO, International Commission on Occupational Health (ICOH) and Finnish Institute of Occupational Health published the BOHS guideline, which provides guidance in the principles, contents, model framework and resources of BOHS in 2005 (1). The BOHS guideline adopts the theory of public health and the principle of primary health care as in the Alma Ata Declaration and the WHO Global Strategy on Occupational Health for All (2, 3). The BOHS guideline supports the ILO Convention No. 155 on Occupational Safety and Health, ILO Convention No. 161 on Occupational Health Services and Occupational Health Services Recommendation No. 171 (4–6).

The concept of BOHS is an essential service in protecting people’s health in the workplace by promoting health, well-being and work capability to prevent unhealthy and accidental injuries. BOHS provide services using appropriate scientific methods and socially acceptable occupational health measures through a primary care approach (1). The main objective of BOHS is to provide OHS to all employees regardless of size, type of industry and geographical location. In other words, it is a universal provision. BOHS was introduced by the Department of Occupational Safety (DOSH) with targeted services to SMEs in Malaysia. The DOSH, in collaboration with the Social Security Organization (SOCSO) and the National Institute of Occupational Safety and Health (NIOSH) Malaysia, also undertakes various programmes for SMEs to improve their working environment, but the accessibility is still low.

About 65.3% of the total employees in Malaysia work in SMEs. Among this percentage, 36.5% is from micro industries, 40.1% from small industries and 23.4% from medium industries (7, 8). Workers in SMEs contribute 36.6% of the gross national product and 18.6% of the country’s exports (7, 8). Various motivational, technical and financial efforts and packages have been provided for SME entrepreneurs to expand and increase their business profits. However, efforts to raise awareness about occupational diseases and assistance in improving the workplace environment to prevent occupational diseases are limited and difficult to observe. Unlike in large and multinational enterprises that usually have in-house OHS, the access of SMEs to OHS is low because of various reasons.

A gap analysis between the current BOHS in Malaysia with the international guideline of BOHS was conducted through a literature review, interviews and meetings with relevant personnel from government and non-governmental institutions and professional bodies in consideration of the current political and social structure and scenarios in Malaysia. One of the important challenges identified that contributes to the low BOHS accessibility in Malaysia is the weakness of the BOHS infrastructure and OHS system provision. In forging ahead to become a developed country, Malaysia requires a comprehensive strategy for the BOHS infrastructure and OHS system provision. The proposed future BOHS infrastructure model ensures that every employee in Malaysia receives OHS.

## Challenges in the BOHS in Malaysia

The BOHS programme recommended by ILO and WHO involves several stages. In stage I, the occupational safety and health (OSH) agents, such as the occupational health nurse (OHN) and the safety and health officer (SHO), conduct a visit to the industry. In stage II, health services are provided to infrastructures near the work areas and the community. Stages III and IV are advanced stages that comprise international standards and comprehensive OHS, respectively (1, 9). The BOHS system involves various activities and processes for the surveillance of both the work environment and the health of workers. According to the conventional model action, the BOHS cycle begins with identifying the needs followed by assessing the problem, taking action, assessing the impact and revising the programme as needed (1).

In Malaysia, the DOSH established its guidelines on OHS in 2005 and the guidelines on BOHS (unpublished) in 2014 (10). The BOHS programme by the DOSH aims to identify the problems related to occupational diseases among workers in SMEs and then to reduce the incidence of occupational diseases and poisoning in Malaysia. The DOSH, with the support of the SOCSO and NIOSH, has also implemented other programmes for SMEs under the strategic OSH Master Plan 2015. The programmes include the Work Improvement in Small Enterprises, Work Improvement for Small Construction Site and Work Improvement and Neighbourhood Development, which all need to be implemented and monitored by state DOSH officers. Also under these programmes are ‘Door to Door’, ‘Umbrella’, ‘Mentor Mentee’, ‘Continuous Registration’, the Campaign Moving Van and the CAT Van (Recognise, Analyse and Action Van) (11–13). These programmes ensure that workers in SMEs have access and support services to implement the BOHS.

The BOHS programme drawn up by the DOSH is diverse and covers almost all aspects of BOHS. The strength of this programme is the surveillance of the work environment. However, the coverage of SMEs that receive BOHS from the DOSH is low. For example, the number of visits of DOSH officers to SMEs in 2015 was only 5,170 and the number of SMEs in Malaysia was 645,136, accounting for a coverage of only 0.8% (14). A significant drawback of the BOHS in Malaysia is the lack of health surveillance activities in SMEs and among self-employed workers. The BOHS programme in Malaysia provides an occupational disease screening tool that is easy to apply by the DOSH officers during their visits to the workplace. Workers suspected of suffering from occupational diseases are referred to a clinic or hospital for diagnosis and treatment once confirmed.

Undoubtedly, health clinics under primary health care, hospitals under the Ministry of Health (MOH) and university hospitals are willing to provide treatment regardless of the type of case, including occupational diseases and poisoning. However, OHS uses the concept of disease prevention and involves different activities other than treatment, such as pre-employment check-ups, return to work programmes and health surveillance, among others, as recommended in the OHS guidelines (10). SMEs, particularly the small and the self-employed, usually face financial constraints, and almost all occupational health clinics are operated by private parties that are commonly concentrated in urban areas. Currently, about 32 occupational health clinics under the management of the MOH provide OHS to MOH employees only. Plans to expand services to other workers have been set up, and they need to be eased, promoted and realised by the MOH.

Generally, primary health care services in Malaysia only focus on treatment and health promotion, and there is no continuity between exposures in the workplace and the health problems suffered by workers. Doctors who treat workers do not have access to the data on chemical health risk assessment and exposure monitoring report in the respective workplaces. Biological monitoring services, medical removal programme, pre-placement assessment and evaluation before retirement are not included in the services offered by primary health clinics in Malaysia. However, pre-employment medical assessment services are available at the MOH primary health clinics, and the services are given according to requirements from employers. The recordkeeping of health workers is similar to that of other outpatients with no specific tagging or history taking on OSH.

In Malaysia, occupational health doctors (OHDs) offer OHS either exclusively in private health clinics or along with general health care services. The demand for health surveillance of workers from SMEs is small. Fees for consulting services are considered expensive and beyond the capacity of SMEs, especially micro and small enterprises. Undoubtedly, there are medium enterprises that have a huge profit margin but lack knowledge and awareness, resulting in their indifference to these legal requirements.

## Proposed Future BOHS Infrastructure Model

Malaysia needs a paradigm shift in terms of BOHS infrastructure to ensure every employer can access OHS. The preparation of the BOHS infrastructure needs to use several models to complement the needs of Malaysia (9). The proposed future BOHS infrastructure model in the near future involves two models, namely, OHD services from private clinics or health centres and group service organisations mobilised by the SMEs themselves. Other models should also be used to ensure that every employee in Malaysia receives OHS. These models include OHS from public primary health care clinics with targeted SMEs, self-employed and civil servants. In-house OHS is a service that works in large enterprises and multinational companies. The advanced reference centre for occupational disease and poisoning in hospitals is a facility that Malaysia needs to prepare for becoming a developed nation. Each unit of this model should be integrated into a comprehensive national OSH system (9).

### Clinic and Private Occupational Health Centres

The OHS model that involves clinics and private occupational health centres is already available in Malaysia. Private clinics with OHDs provide OHS and primary health care services to the community, and private occupational health centres focus on OHS. However, most industries that use these services are large- and medium-sized enterprises. They usually have an in-house SHO and appoint an OHD as a panel doctor or a visiting doctor in the company. The involvement of micro- and small-sized enterprises in this OHS model is limited because of financial factors.

### OHS Practitioner Group Centre

OHS practitioner group models practicing near SMEs can be encouraged and publicised. The establishment of an OHS practitioner group is the initiative of several employers hiring a group of consultants in the field of OSH on a part-time basis. The main OHS practitioner group members are the OHN, SHO and OHD. Alternatively, other OHS practitioner groups may consist of hygiene technicians, occupational hygiene experts, ergonomists and rehabilitation officers. Members of the BOHS practitioner group are co-utilised by the industries that hire them. Such shared systems should lead to economies of scale in which the cost of employing OSH agents becomes less expensive. The responsible party for developing the industrial area, especially the SMEs, should also provide infrastructure for the OHS practitioner group centre aside from providing basic infrastructure, such as industrial buildings, roads, safety controls and fire services.

### Public Primary Occupational Health Clinic

A significant weakness in the delivery of the proposed BOHS programme for immediate implementation is the cost of sending employees to a private health clinic or an OHS practitioner group centre, which will be fully borne by the employer. The capability of SMEs, especially micro and small enterprises, and that of the self-employed to pay for external services are low or may not exist at all. Only 2.3% of SMEs, which account for 23.4% of jobs in Malaysia from the medium industry group, are expected to benefit from this system (7, 8). About 97.7% of SMEs (21.2% of small industry businesses and 76.5% of micro industry businesses), which account for 76.6% of the total occupation, are unlikely to benefit from this system (7, 8). Through this, the provision of the BOHS programme infrastructure for the self-employed and the micro and small industry sectors should be guaranteed by the government through the delivery of government primary health care services. For Malaysia to provide a comprehensive OHS, public primary health care clinics should also provide OHS to their 1.6 million civil servants. The current OHS provision for MOH workers is a good beginning and should be strengthened.

According to the ILO Convention No. 161 on OHS, the responsibility of providing OHS lies with the employer. However, the BOHS programme should also be viewed from the point of responsibility of the authorities providing the public health services to the community. The BOHS programme policy came from the public health policy, the ‘Health for All’ strategy in 1977, and was further supported by the Alma Ata Declaration in 1978 (2). The Alma Ata Declaration emphasised a primary health care with a national health system that brings health care close to where the people live and work. The WHO’s 2000 Health for All global strategy aimed at making the health infrastructure part of primary health care by emphasising the health system, which comprises the components associated with residence, educational institutions, workplaces, communities and the health sector (15). Occupational health is one of the key elements in implementing the ‘Health for All’ policy recommended by the WHO.

In accordance with the strategic planning of the BOHS programme recommended by the WHO/ILO/ICOH, the cooperation of the Ministry of Human Resources (MOHR) and the MOH at the national level is necessary for our country to achieve the BOHS programme objectives. For example, Japan and Finland have a safety and health department under one ministry, thus making the implementation of the OHS program more efficient. Taiwan, similar to Malaysia, has safety and health departments under different ministries but has succeeded in establishing cooperation and enhanced OHS coverage to about 20% (16). The Taiwanese government founded the Network of Occupational Diseases and Injuries Service and established a new Internet-based reporting system, which increased the OHS accessibility and notification of occupational disease and injuries (17). Such collaboration with distinctive models and modules is necessary to ensure that all Malaysians obtain a fair and comprehensive health service.

The SOCSO has been involved in different disease and workplace accident prevention and rehabilitation programmes for Malaysian workers. It is also about time that the SOCSO runs its occupational health clinic to meet the government’s goal of protecting workers, especially those in SMEs, from occupational disease and poisoning. This investment should benefit the SOCSO in the long run. The strategy for the construction and provision of the SOCSO’s occupational health clinic should be in accordance with their financial assistance strategy.

### Tertiary Referral Centre for Occupational Disease and Poisoning

The future for the BOHS programme should involve the development of a tertiary referral centre for occupational disease and poisoning. This strategy may be achieved with the cooperation of university training hospitals that have expertise in occupational health. It can be initiated by referring occupational disease and poisoning to the referral centres, and the services can be expanded by adding specialists to the team. The specialists should consist of physicians, orthopaedic surgeons, dermatologists, rehabilitation specialists, psychologists and psychiatrists who had undergone occupational health training. The final goal of the programme is to provide hospital beds for occupational disease and poisoning cases. This approach uses existing university hospitals and clinical infrastructure, empowers existing expertise and encourages research development in occupational health and occupational medicine. The proposed future model of OHS in Malaysia, the involvement of OSH agents and their industrial targets are shown in Table 1.

### Occupational Health Service Integration System

Each unit of the model should be integrated into a comprehensive national OSH system (9). Such integrated systems not only involve infrastructure from various ministries, departments and agencies but also require an efficient and effective communication system to ensure the smooth implementation of the programme. An Occupational Health Committee under the National Council for Occupational Safety and Health has already been established, and it consists of three important parties, namely, the government, the employers and the employees, and the other parties including the MOH, NIOSH, SOCSO and other relevant professional bodies and social associations. The Ministry of Higher Education and the Ministry of Communication and Multimedia are suggested to be included as members in the committee to facilitate the establishment of a tertiary referral centre for occupational diseases and poisoning in university hospitals and to enhance the promotion of the BOHS programme nationwide. In particular, the committee shall coordinate and integrate BOHS activities, review the achievement and take necessary action for improvement.

## Conclusion

The DOSH faces great challenges in its delivery of OHS and BOHS because of the constraints of the OHS infrastructure and system. To overcome these challenges, Malaysia requires a paradigm shift in terms of infrastructure provision and OHS system. These approaches need to be spearheaded by the government, which should clearly outline the roles and responsibilities of its machinery. The MOHR and the MOH are the main machineries in the implementation of the BOHS, and they need to work closely and be responsible for integrating the entire infrastructure and BOHS programme activities.

## Figures and Tables

**Table 1:** The proposed basic occupational health services (BOHS) infrastructure model for Malaysia

Model	OSH Agents	Target Groups
Private clinics	OHD	Large and medium enterprises (high risk manufacturing: > 100 employees), large enterprise (other than manufacturing sector: > 500 employees)
Private occupational health clinic	OHN, OHD, ergonomist, rehabilitation officer, hygiene specialist, SHO and others.	
OHS group practice centre	OHN, SHO, OHD, ergonomist, hygienic specialist, and others.	Small enterprise (manufacturing: 5–75 employees; other sector 5–30 employees), medium enterprises (manufacturing: 75–200 employees; other sector 30–75 employees)
Public occupational health clinic	OHN, OHD, SHO	Self-employed, micro enterprise (< 5 employees), small enterprise (manufacturing: 5–75 employees; other sectors 5–30 employees), public servant
SOCSO occupational health clinic	OHN, SHO, OHD, ergonomist, rehabilitation officer, hygiene technician and others	Self-employed, micro enterprise (< 5 employees), small enterprise (manufacturing: 5–75 employees; other sector 5–30 employees)
In-house OHS	OHN, OHD, SHO	Large enterprise and multinational (> 1000 employees)
Tertiary referral centre	OHN, OHD and medical specialist, orthopaedic, skin, psychiatry, rehabilitation officer with training in OSH field	All

OHN (Occupational Health Nurse), SHO (Safety and Health Officer), OHD (Occupational Health Doctor), OHS (Occupational Health and Safety), SOCSO (Social Security Organization)
